# International Congress of Hygiene and Demography

**Published:** 1894-09

**Authors:** W. H. Dalrymple

**Affiliations:** M.R.C.V.S. (Eng.); Late Veterinarian, Louisiana State Bureau of Agriculture; Atlanta, Ga.


### INTERNATIONAL CONGRESS OF HYGIENE AND DEMOGRAPHY.

### SECTION XVIII.

It may be of interest to the readers of THE SOUTHERN MEDICAL RECORD to have brought before their notice the particulars of this section, which is confined to veterinary medicine, and which is to occupy, along with others, the attention of the Eighth International Congress of Hygiene and Demography, to be held at Budapest, in Hungary, during the first nine or ten days of September. The following are the subjects to be discussed, and it will be observed that a number of them are not only of interest and importance to the veterinarian, but we might venture to say, even more so to the practitioner of human medicine, as they affect, to a very large extent, a nation's health.

1. Preventive inoculation against anthrax and swine-plague.
2. Preventive inoculation against bovine pneumonia.
3. Inoculation against symptomatic anthrax.
4. Diagnostic value of tuberculin.
5. Diagnostic value of mullein.
6. The pneumo-bacillus and pneumo-bacillin.
7. Organization for meat inspection.
8. Regulation of the production of milk from a sanitary point of view.
9. Disease producing parasites.
10. Means of preventing the propagation of tuberculosis.
11. Prophylactic measures against aphthous fever.

In our more southerly States, the veterinary profession has not as yet attained to the prominent position amongst the learned profession to which we hope some day, not in the very far distant future, to see it ranking as an indispensable and necessary branch of medical science.

To a great majority of the people the term veterinary surgeon is simply a synonym for the "old-time horse doctor," or empiric, with his quackish nostrums and superstitious absurdities, compounding his specifics at certain phases of the moon, and performing his so-called operations under similar conditions. Men of this class are still to be found in all civilized countries, not altogether allied to the veterinary profession. But the day is fast approaching, in fact is now with us, when none but those thoroughly educated and trained will have any chance of keeping abreast of veterinary medicine on account of the rapid progress it has been making in the last twenty years.

The veterinary field to-day is an exceedingly wide and important one (a fact which is often lost sight of by many), having, from the the standpoint of national progress and public welfare, played a most important part in the past, and is destined to greater achievements in the future.

In the Northern and Eastern States of the Union and in European countries, the veterinary profession is recognized, presumably because, as a profession, its value is better known and appreciated.

In a recent address given to the students of the Royal (Dick) Veterinary College, Edinburgh, Scotland, by John Chiene, M.D., Professor of Surgery in the University of Edinburgh, the professor addressing his auditors, remarked:

"Fellow students of medicine in the widest sense of the term, the main reason I am here is that I am anxious to impress upon you to-day the close relationship between your work and mine, and I wish to take as an illustration an important zoögenous disease, because in it we have a common enemy, against whose evil influences we have to be constantly on the watch. I refer to tuberculosis."

In another passage, the professor remarks: "I am here to-day to intensify the opinion that we exist as a mutual help to one another. As a surgeon I wish your help as veterinarians, in the battle with the tubercle fiend."

In his work on "Meat Inspection," Professor Walley, M.R.C.-V.S., Edinburgh, says: "Seeing that the primary aim of the members of both professions is to conserve the human and animal populations, it is surely not too much to ask that they should work in unison to accomplish the desired end."

William Hunting, Esq., F.R.C.V.S., President of the Royal

College of Veterinary Surgeons of England, in an address before the National Veterinary Association recently, said: "So far, gentlemen, I have referred only to the part of the work we have done in protecting the public health. I now desire to make a claim that we have protected the public *health*, and that we are in a position to assist the medical officer to an extent quite unappreciated by the public, but now handsomely acknowledged by the medical profession."

The diseases of animals that are communicable to man are an important class, and the protection of human life requires that action against them should be taken before they pass from the animal. It is for veterinarians to diagnose them in their patients, and then to take such measures as may prevent their spread.

Rabies, anthrax, and glanders never affect human beings except when the specific virus is transmitted from animals either directly or indirectly. The bacillus of tuberculosis may be conveyed either by milk or flesh.

Various parasitic diseases can only arise in man after they have existed in animals. Every advance in veterinary science which lessens the prevalence of these transmissible diseases is a further protection to human life.

It has been well said that there is only one pathology, but the subject is so vast that its departments must be intrusted to specialists. The study of disease embraces the morbid conditions found in man, in animals and in plants. No one mind can master the subject in every section, and yet pathological science depends for its fullest development upon comparing the results of research in every direction.

To the veterinarian must be intrusted a large share in the investigation of animal maladies.

The experience of the veterinary pathologist is a necessary corollary to the experience of the human pathologist. Sometimes the former can add to the general knowledge, and sometimes he may correct the errors which so easily beguile the investigator unaccustomed to the diseases of the lower animals.

From the report recently published of the proceedings of the National Live Stock Sanitary Convention held in Washington City,

D. C., in June last, we find that the main object of the convention was to discuss and legislate measures in the interests of the public health, as well as that of the lower animals, as will be seen by a glance at the subjects such as, "Uniform State Laws for the Suppression and Prevention of the Spread of Contagious and Infectious Diseases among the Domestic Animals," "Actinomycosis," "Tuberculosis," "Boards of Health," "Inspection of Animals," "Slaughter Houses," etc.

Among those present at the convention was Dr. D. E. Salmon, Chief of the United States Bureau of Animal Industry, as well as other eminent veterinary authorities from all over the Union.

Our object in penning this short article is, as we mentioned at the beginning, to bring before the readers of THE RECORD a program which we feel sure will interest them from the standpoint of comparative medicine, as well as to touch upon the veterinary profession, the capabilities of which do not seem to be realized, especially in our more southerly section of the Union.

It must, however, appear evident that the work in transmissible diseases alone, which affect both branches of the medical profession, is great, and that sympathy and co-operation in such work could not result otherwise than in the public good.

W. H. DALRYMPLE, M.R.C.V.S. (Eng.),
Late Veterinarian, Louisiana State Bureau of Agriculture.
*Atlanta, Ga.*

